# The Effects of an Oral Supplementation of a Natural Keratin Hydrolysate on Skin Aging: A Randomized, Double‐Blind, Placebo‐Controlled Clinical Study in Healthy Women

**DOI:** 10.1111/jocd.16626

**Published:** 2024-10-04

**Authors:** Francesco Tursi, Vincenzo Nobile, Enza Cestone, Ileana De Ponti, Anne Lepoudere, Renaud Sergheraert, Jean‐Philippe Soulard

**Affiliations:** ^1^ Clinical Testing Department Complife Italia S.r.l. San Martino Siccomario PV Italy; ^2^ R&D Department Complife Italia S.r.l. San Martino Siccomario PV Italy; ^3^ R&D Department BCF Life Sciences Pleucadeuc France

**Keywords:** aminobiotics, anti‐aging, extensive hydrolysis, free amino acids, keratin hydrolysates

## Abstract

**Background:**

Keratin hydrolysates are active components used in food supplements to alleviate aging signs on skin, hair, and nails.

**Aims:**

This randomized, double‐blind, placebo‐controlled study evaluates a novel keratin hydrolysate obtained from poultry feathers. This feather keratin hydrolysate (FKH) results in a characteristic mix of free L‐amino acids (≥ 83.5%). FKH was administered as a food supplement to a panel of adult women showing aging physiological signs.

**Methods:**

Participants were randomly assigned in three groups to receive daily dosages of 500 or 1000 mg of FKH or placebo for 90 days. Parameters of skin roughness, wrinkle features, deep skin moisturization, skin maximum elongation and elasticity, skin thickness, skin anisotropy, skin density, gloss of skin, hair and nails, and nail hardness were evaluated. Subjects also answered a questionnaire related to the treatment efficacy perception.

**Results:**

Both FKH treatments showed a significant improvement of all parameters compared to day 0 and to placebo, with an exception for fiber anisotropy and fiber density which showed a significant improvement compared to day 0 and a tendency to improve compared to placebo. These measurements were bolstered by the results of a self‐assessment questionnaire, showing an overall set of positive answers for both treatments compared to placebo.

**Conclusions:**

Oral supplementation of FKH for 90 days is associated with an improvement in the appearance of facial skin, hair, and nails. This study highlights the benefits of free L‐amino acids mix as potential aminobiotics and not just as building blocks of proteins, suggesting a new perspective of nutricosmetic food.

## 1 Introduction

The skin is the largest organ of the human body and plays a vital role in several dynamic processes that contribute to homeostasis [1]. Our bodies face a complex task each day in maintaining and re‐establishing skin homeostasis. Skin is characterized by a high proliferation rate, and its functioning is based on a precarious balance prone to external disturbances. Clinical signs of skin aging can be explained by an overall disturbance in this well‐balanced functionality. This natural and complex process is influenced by two mechanisms: intrinsic aging (genetic and chronological) resulting from the passage of time and extrinsic aging caused by environmental factors such as UV radiation (photoaging), environmental pollution, unbalanced diet, and cigarette smoke [2]. Features of mature skin include wrinkles, dryness, loss of elasticity and firmness, changes in color, uneven pigmentation, susceptibility to irritation, and slower skin regeneration and healing [2].

Whereas topical cosmetic treatments have often been the first approach to counteract, improve, and/or mask skin aging signs, food supplements represent a promising alternative approach. Providing the body with highly efficient and selective nutrients, food supplements could be directed toward a wider range of biological targets, resulting in an optimization of cellular regeneration, reestablishing skin homeostasis, as well as slowing down, and preventing signs of aging [3, 4].

Nutrient and nonnutrient ingredients obtained from diet or oral supplementation have already been recognized for their positive effects on the appearance of skin through antioxidant, hydrating, photoprotective, or anti‐inflammatory actions [5]. Among these solutions, essential fatty acids, coenzyme Q10, curcumin, polyphenols, flavonoids, probiotics, amino acids, and various bioactive peptides such as collagen‐derived peptides have been investigated for their skin, hair, and nail beauty benefits [6].

While there are multiple published studies on the use of nutritional supplements for improvement of various skin or hair conditions, most of them evaluated complete formulas including active ingredients but also vitamins and minerals. Moreover, the rational chain of scientific evidence is frequently insufficient, and few studies are designed to the gold standard of a randomized, double‐blind, placebo‐controlled trial and, as a result, lack scientific credibility [4, 7].

Keratin is among the most abundant structural proteins in humans and animals and is a potential source of variety of free amino acids and biopeptides. Different kinds of keratins have been studied for their benefits on hair, especially for temporary hair loss (acute telogen effluvium), and for nail brittleness [8, 9, 10]. However, the role of keratin supplementation in preventing skin aging remains broadly understudied [11].

Keratin is a fibrous and rigid protein that forms the structural basis of various tissues including skin, hair, and nails [12]. This protein is exclusively produced by animals, and is the most abundant biomaterial after collagen. Unfortunately, due to its molecular structure, this protein is not metabolized by mammalian enzymes and thus has a very low nutritive value. The bioavailability of keratin for humans is close to zero [13]. Acid hydrolysis is the most efficient method for breaking down keratin into free amino acids of L‐form and small peptides (< 800 Da). The composition of hydrolysates obtained through this process depends on various parameters. Kera‐Diet (sold as KeraGLO in the United States) is a patented keratin‐hydrolysate solution [14] that contains a high concentration of free amino acids (≥ 83.5%) and is highly digested.

Amino acids are the basic building blocks of peptides and proteins, and they play a vital role in cellular signaling function in human biology [15, 16]. The benefits of amino acids supplementation have already been studied in different animal models, and results show a restoration of skin damage, an improvement of skin hydration, wound healing, as well as protein metabolism which leads to collagen synthesis [16, 17].

The current randomized, double‐blind, placebo‐controlled clinical trial evaluates for the first time the efficacy of the hydrolyzed keratin‐based ingredient Kera‐Diet as a unique active ingredient in adult women showing physiological aging signs on skin, hair, and nails using clinical grading, bioinstrumentation, and subject self‐assessments.

## 2 Materials and Methods

### 2.1 Tested Products, Randomization, and Compliance

The tested food supplement, feather keratin hydrolysate (FKH) commercialized under the brand name Kera‐Diet, BCF Life Sciences, Boisel, Pleucadeuc, France (also, KeraGLO, NutriScience Innovations, Milford, Connecticut, USA), is a hydrolysate of natural keratin obtained from poultry feathers through a patented extensive hydrolysis process [14].

**FIGURE 1:**
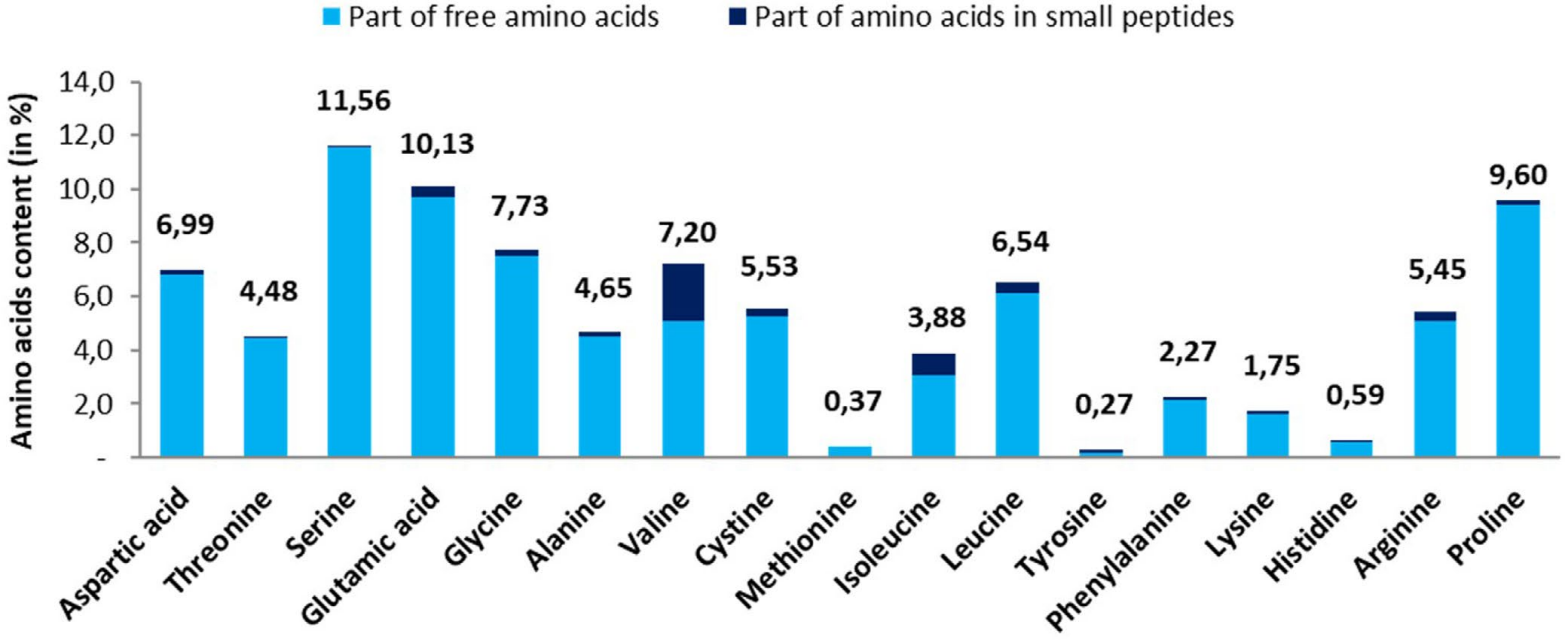
Amino acids profile expressed in amino acid content (in % w/w) of FKH product.

FKH is mainly composed of free amino acids (Figure 1) and has a similar profile to hair keratin. It is characterized by a stable and unique profile of 17 amino acids, at least 83.5% of them in free form, and by a high absorption due to a very low molecular weight (100% of the product is under 800 Da).

Capsules containing two dosages of FKH (125 and 250 mg) plus maltodextrin and magnesium stearate were manufactured using maltodextrin for adjusting the weight as well as for preparing placebo capsules.

FKHs and placebo were randomly allocated by a computer‐generated (PASS 11, version 11.0.8; PASS, LLC. Kaysville, UT, USA) restricted and balanced (1:1:1) randomization list created by the “Wey's urn” algorithm. The randomization list was then concealed in opaque envelopes and ordered by a sequential number. Separation was maintained between the investigator and the study staff that delivered the tested products.

Compliance with treatments was assessed by counting and recording the remaining capsules at each checkpoint. The threshold value for compliance with treatments was set at ≥ 80%.

### 2.2 Study Design

In this multicentric, randomized, double‐blind, placebo‐controlled study, each participant took four capsules per day (two at breakfast and two at dinner) of the placebo or the test product with a glass of water for 90 days, providing a dosage of 500 or 1000 mg of FKH per day.

Safety and efficacy assessments included three visits: a baseline visit (D0), an intermediate visit after 45 days (D45), and a final visit after 90 days (D90) of product intake. To standardize the test conditions, volunteers were supplied with a face base (i.e., without any specific/peculiar cosmetic activity) cream to be used during the whole study period instead of their day/night face cream. Volunteers were instructed to not apply the cream in the morning of each visit.

The primary objective of this study was to evaluate the maintenance of skin homeostasis and the anti‐aging/moisturizing efficacy of the tested products. Specifically, skin wrinkles, skin roughness, skin firmness and elasticity, skin gloss, deep skin moisturization, skin thickness, and skin fiber network were evaluated. Secondary objectives of this study were to evaluate the effect of the tested products in improving hair gloss, nail gloss, and nail hardness/aspect to confirm the efficacy observed in an initial study published in 2019 and 2021 [9, 10].

The study took place at Complife Italia Srl facilities in Italy (Pavia and Biella). All the study related procedures were carried out in accordance with the Declaration of Helsinki. Study protocol and the Informed Consent Form (ICF) were approved by the “Independent Ethical Committee for Non‐Pharmacological Clinical trials” Genova, Italy (ref. 2022/04). Before the initiation of any study‐related procedures, all the subjects were fully informed of the study risks and benefits, aims, and procedures, and they provided a written ICF and signed a photo consent. Study was registered at ISRCTN registry (Registration number: ISRCTN10686369, https://doi.org/10.1186/ISRCTN10686369).

### 2.3 Study Participants

Ninety‐nine (*n* = 99) healthy women were enrolled by dermatologists according to the following inclusion criteria: subjects aged between 35 and 65 years old (50.4 ± 0.7), phototypes I–IV (according to Fitzpatrick classification), clinically showing physiological aging signs, visible face roughness (crow's feet wrinkles), mild to moderate skin sagging, damaged/brittle hair, and brittle nails. Exclusion criteria were as follows: pregnancy or intention to become pregnant, lactation, subjects with a consumption of food supplement(s) containing active ingredients having an influence on skin/hair/nail care on‐going or within the past 12 weeks before the study, and any skin care‐make‐up/hair care product applied on the skin in the 3 h before the visit and on scalp between the last shampoo and the inclusion visit (e.g., gel, hairspray, wax, foam, …) and sun exposure (both natural and artificial) 2 months before the study start. Intensive exposure to UV rays and hair dying 3 weeks before each follow‐up visits were prohibited. Subjects were asked to use the same shampoo during all the study period.

### 2.4 Measurements

Clinical and instrumental evaluations were carried out under temperature and humidity‐controlled conditions (temperature: 22°C ± 2°C and relative humidity: 50% ± 10%) after a 15–20 min acclimatization period.

Skin profilometry was evaluated on the periorbital area using PRIMOS^CR^ (Canfield Scientific Europe, BV, Utrecht, the Netherlands). PRIMOS^CR^ is an optical three‐dimensional noncontact skin measurement device based on structured light projection that allows to measure skin surface properties. In this study, skin roughness and skin wrinkles depth, length, and area were measured in the “crow's feet” area.

Skin, hair, and nail gloss were measured as the 8° gloss value (specularly reflected light) by using a spectrophotometer/colorimeter CM‐700D (Konica‐Minolta, Italy). The gloss value is used in the management of the brilliance of the color.

Deep Skin Moisturization of cheeks was measured by the MoistureMeterEpiD (Delfin Technologies BG, Italy). The device is an all‐in‐one measurement unit (composed of an integrated probe, a built‐in contact force sensor and a display) that allows a noninvasive measurement of the percentage of local tissue water (0%–100%) in the skin (500 μm).

Skin elasticity and firmness were evaluated using a Cutometer MPA 580 (Courage + Khazaka Electronic GmbH, Köln, Germany). Skin elasticity, which represents the ability of the skin to return to its original state after a stressing event, was calculated as the R2 parameter, that is, the ratio between the residual deformation (Ua) and the maximum elongation of the skin (Uf). Skin firmness was calculated as the R0 parameter and indicates the ability of the skin to oppose the deformation.

Skin thickness (of the epidermis) was measured on transversal picture taken at the cheeks level using the DeepLive (DAMAE Medical, Paris, France) device which integrates LC‐OCT technology (Line‐field Confocal Optical Coherence Tomography). LC‐OCT technology was also applied for measuring the overall fiber anisotropy (measuring global orientation of fibers calculated from the polar distribution diagram) and fiber density (which reflects the number of fibers). The fiber analysis was performed on horizontal images at a fixed distance from the dermal‐epidermal junction. The dermal fibers are automatically segmented on the horizontal images [18, 19].

Nail hardness/aspect was clinically assessed according to a visual analogue scale (VAS) from 0 to 10, where: 0 = very fragile nails that flake and break easily and with irregular margins and 10 = very hard nails, which do not break and do not flake and with very regular margins.

At the end of the trial, subjects were asked to answer a self‐assessment questionnaire referring to the perceived effect of the treatment on skin (16 questions), hair (5 questions), and nails (5 questions) as well as on 3 questions referring to the global acceptance of the products (continue to use, intention to buy, recommendation).

### 2.5 Statistical Analysis

Sample size was calculated with a two‐sided 5% significance level and a power of 80% considering a 12% variation of the primary endpoint (wrinkle depth) due to both inter‐individual human variability and error in the measurement techniques. A sample size of 27 subjects per group was obtained using PASS 11 statistical software (version 11.0.8 for Windows) running on Windows Server 2008 R2 Standard SP1 64‐bit edition (Microsoft, USA).

The dropout rate was estimated, at 20% and thus, the initial sample size for each group was fixed at 33 persons.

Results, graphs, and statistical analyses refer to per‐protocol population (PP). Missing data (i.e., drop‐outs) were not considered in the analysis.

An appropriate statistical model was applied based on data distribution as follows:

‐ 2‐way Student's *t*‐test for paired and normally distributed data (intra‐ and inter‐group statistical analyses);

‐ Wilcoxon signed rank test for paired and not normally distributed data (intra‐group statistical analyses);

‐ Mann–Whitney *U*‐test (Wilcoxon Rank Sum test) for not normally distributed data (inter‐group statistical analyses).

Student's *t*‐test was carried out using a Microsoft Excel worksheet. The statistical software used for the other statistical analyses was NCSS 10 (version 10.0.7 for Windows; NCSS, Kaysville, UT, USA).

## 3 Results

**FIGURE 2:**
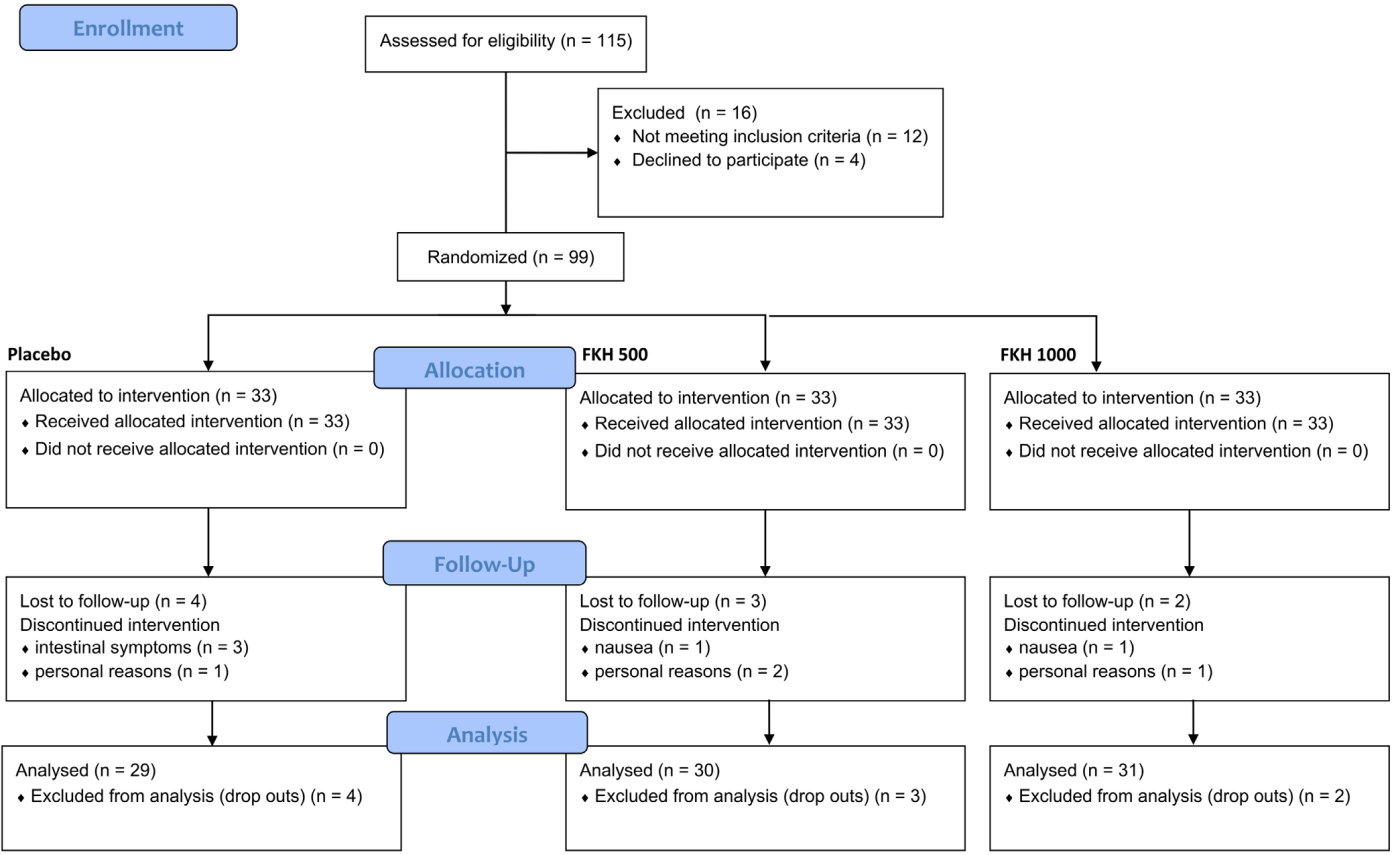
Participant flow chart.

The study was conducted between July 2022 and May 2023. A total of 99 Caucasian women were successfully enrolled and randomly assigned to the three groups (Figure 2). No significant differences were recorded in the baseline characteristics of measured parameters across treatment arms, indicating unbiased randomization and the absence of covariates.

Product intake was well tolerated. Nine subjects dropped out, as follows: four subjects from the placebo group, three subjects from the 500 mg FKH group, and two subjects from the 1000 mg FKH group. Four subjects dropped out for personal reasons not related to the product intake, the remaining dropouts shared intestinal problems, probably related to seasonal flu. Efficacy analysis was carried out on the per protocol population on 29 subjects in the Placebo group, on 30 subjects in the 500 mg FKH group, and on 31 subjects for 1000 mg FKH group.

All subjects were compliant with the treatment scheme, and no major or minor protocol deviations were recorded in the treatment regimen for all the study groups. The compliance with treatment was > 90%.

### 3.1 Skin Profilometry

**FIGURE 3:**
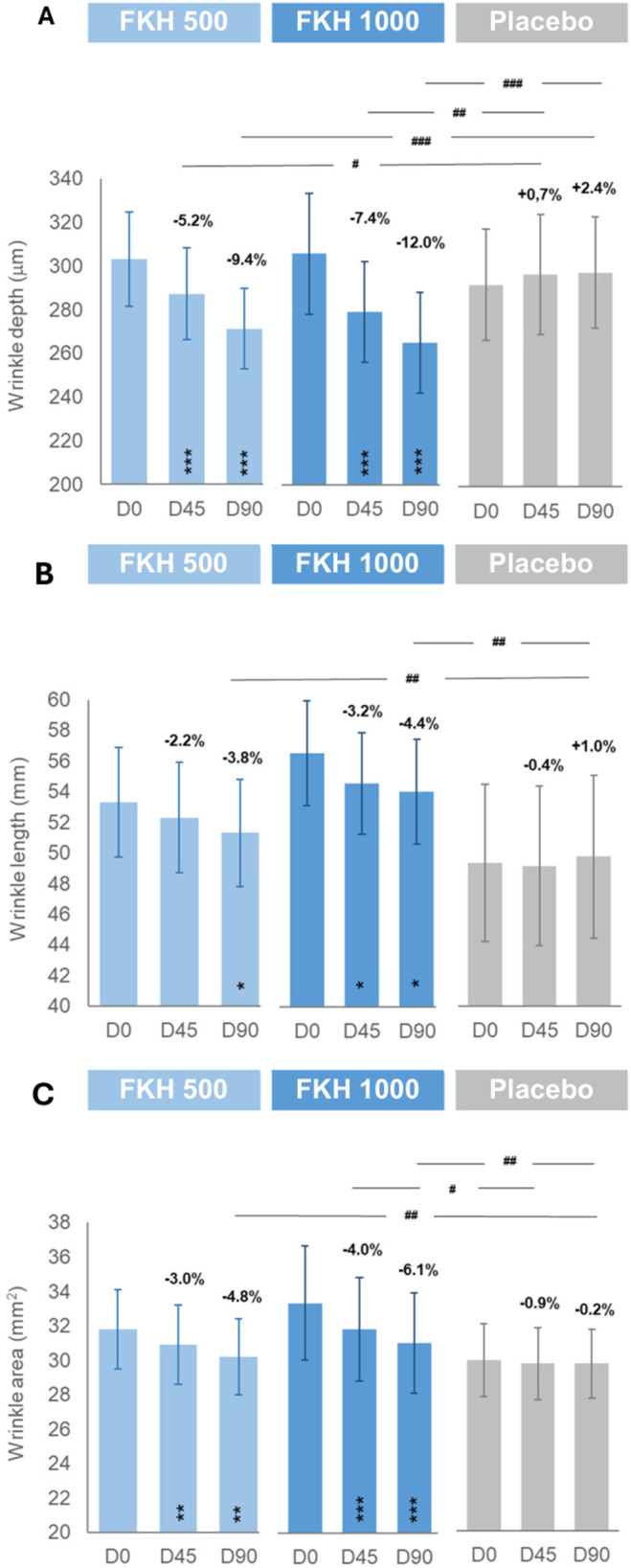
Effect of treatments on wrinkle depth (A), wrinkle length (B), and wrinkle area (C). Results are expressed as mean ± SEM of the respective parameters and as % of variation versus D0 (Δ%45 or Δ%90). Intragroup* and intergroup^#^ (actives vs. placebo as Δ%45 or Δ%90) statistical analyses are reported as *^,#^ *p* < 0.05, **^,##^ *p* < 0.01, and ***^,###^ *p* < 0.001.

FKH treatments resulted in a progressive reduction of skin roughness, inversely related to skin smoothness (Table 1), as well of wrinkle depth, length, and area (Figure 3) in the “crow's feet” area. The improvement was statistically significant at D90 versus D0 for all parameters and at D45 versus D0 for skin roughness, wrinkle depth, and wrinkle area, whereas placebo treatment did not show any relevant changes in the investigated parameters between D0 and D90 throughout the study.

**TABLE 1:** Skin roughness.

	D0	D45	Δ%45	D90	Δ%90
Skin roughness (μm)					
Placebo	29.0 ± 0.8	29.3 ± 1.0	+0.9%	29.5 ± 0.8	+2.0%
FKH 500	30.3 ± 1.0	29.2 ± 0.9**	−3.4%^#^	28.2 ± 0.9***	−6.4%^###^
FKH 1000	29.9 ± 1.3	28.3 ± 1.1***	−4.6%^#^	27.6 ± 1.1***	−7.2%^###^

*Note:* Intragroup* statistical analyses are reported next to raw data (mean ± SEM); intergroup^#^ (actives vs. placebo) and intergroup ʃ (between actives) statistical analyses are reported next to the % variation versus D0 (Δ%45 or Δ%90) and are calculated as average of the variation of each subject. Statistical analyses are reported as follows: *, #, ʃ *p* < 0.05, **, ##, ʃ ʃ *p* < 0.01, and ***, ###, ʃ ʃ ʃ *p* < 0.001.

The active treatments showed a progressive reduction in terms of mean percentage variation of skin roughness (−3.4% ± 1.0% and − 4.6% ± 1.4% at D45, −6.4% ± 1.2% and − 7.2% ± 1.6% at D90 for FKH 500 mg and FKH 1000 mg, respectively), wrinkle depth (−5.2% ± 1.1% and − 7.4% ± 1.4% at D45, −9.4% ± 1.6% and − 12.0% ± 1.9% at D90 for FKH 500 mg and FKH 1000 mg, respectively), wrinkle length (−3.8% ± 1.4% and − 4.4% ± 1.4% at D90 for FKH 500 mg and FKH 1000 mg, respectively), and wrinkle area (−3.0% ± 0.9% and − 4.0% ± 1.0% at D45, −4.8% ± 1.2% and − 6.1% ± 1.4% at D90 for FKH 500 mg and FKH 1000 mg, respectively), which resulted in a significant intergroup reduction compared to placebo for all treatments except for wrinkle length at D45 and wrinkle area at D45 for FKH 500 mg. Although FKH 1000 mg treatment achieved a higher improvement with respect to FKH 500 mg, no intergroup significant difference between the active treatments was observed throughout the study.

### 3.2 Deep Skin Moisturization

All treatments showed a progressive improvement of the initial facial deep skin moisturization (Table 2), indicating an increase of the percentage of water contained in the epidermis. The increase was significant for both FKH treatments throughout the study and for the placebo treatment at D90. Highest mean percentages of variation versus D0 were achieved at D90 by FKH 500 mg and by FKH 1000 mg, +11.1% ± 1.8% and + 17.0% ± 2.1%, respectively, which resulted in a significant intergroup difference with respect to placebo and in a significant difference between the two active treatments (*p* < 0.05).

### 3.3 Skin Elasticity and Skin Firmness

FKH treatments showed a progressive and significant intragroup improvement in facial skin elasticity (R2) and a significant reduction of the maximum elongation (R0). Skin firmness, defined as inversely proportional to R0, significantly improved from D45, whereas no appreciable change was achieved by the placebo treatment between D0 and D90 (Table 2).

**TABLE 2:** Skin parameters.

	D0	D45	Δ%45	D90	Δ%90
Deep moisturization (%)					
Placebo	35.7 ± 2.0	36.1 ± 1.7	+2.7%	36.7 ± 1.9*	+4.0%
FKH 500	39.4 ± 1.5	42.4 ± 1.6***	+8.0%	43.6 ± 1.6***	+11.1%^##^
FKH 1000	35.1 ± 1.9	37.8 ± 1.8***	+9.2%^#^	40.8 ± 2.1***	+17.0%^### ʃ^
Skin elasticity, *R*2 (ratio)					
Placebo	0.6377 ± 0.0091	0.6399 ± 0.0093	+0.4%	0.6410 ± 0.0091	+0.6%
FKH 500	0.6460 ± 0.0127	0.6664 ± 0.0110***	+3.4%^###^	0.6820 ± 0.0121***	+5.8%^###^
FKH 1000	0.6305 ± 0.0100	0.6502 ± 0.0105***	+3.1%^###^	0.6782 ± 0.0122***	+7.6%^###^
Skin maximum elongation, *R*0 (mm)					
Placebo	0.3898 ± 0.0098	0.3853 ± 0.0101	−1.2%	0.3863 ± 0.0098	−0.9%
FKH 500	0.3968 ± 0.0108	0.3726 ± 0.0103***	−6.0%^###^	0.3575 ± 0.0106***	−9.8%^###^
FKH 1000	0.3772 ± 0.0110	0.3552 ± 0.0117***	−5.7%^#^	0.3406 ± 0.0115***	−9.7%^###^

*Note:* Intragroup* statistical analyses are reported next to raw data (mean ± SEM); intergroup^#^ (actives vs. placebo) and intergroup ʃ (between actives) statistical analyses are reported next to the % variation versus D0 (Δ%45 or Δ%90) and are calculated as average of the variation of each subject. Statistical analyses are reported as follows: *, #, ʃ *p* < 0.05, **, ##, ʃ ʃ *p* < 0.01, and ***, ###, ʃ ʃ ʃ *p* < 0.001.

In terms of mean percentage of variation versus D0, both active treatments showed a progressive improvement in skin elasticity (+3.4% ± 0.8% and + 3.1% ± 0.6% at D45, +5.8% ± 0.8% and + 7.6% ± 1.0% at D90 for FKH 500 mg and FKH 1000 mg, respectively) and a progressive reduction of skin maximum elongation (−6.0% ± 1.1% and − 5.7% ± 1.5% at D45, −9.8% ± 1.4% and − 9.7% ± 1.5% at D90 for FKH 500 mg and FKH 1000 mg, respectively), which resulted in a significant intergroup improvement achieved by all treatments in comparison with the placebo. No intergroup significant difference between the active treatments was observed throughout the study.

The results indicate that treatments with natural keratin highly concentrated in free amino acids improve skin elasticity and firmness.

### 3.4 Gloss Parameter

FKH treatments showed a progressive and significant intragroup improvement in gloss parameters measured on the skin face as well as on the hair and on the nails (Table 3). Placebo treatment showed a slight improvement in initial values of all gloss parameters, with significance only for hair at D90.

**TABLE 3:** Gloss parameters.

	D0	D45	Δ%45	D90	Δ%90
Skin gloss (a.u.)					
Placebo	11.18 ± 0.61	11.17 ± 0.56	0.8%	11.30 ± 0.50	2.8%
FKH 500	10.58 ± 0.74	13.09 ± 0.83 ***	26.5%^###^	13.85 ± 0.79 ***	35.8%^###^
FKH 1000	10.31 ± 0.66	12.63 ± 0.78 ***	25.3%^###^	14.33 ± 0.82 ***	43.5%^###^
Hair gloss (a.u.)					
Placebo	4.89 ± 0.47	5.04 ± 0.44	9.1%	5.28 ± 0.48 *	10.8%
FKH 500	7.14 ± 1.02	8.24 ± 1.03 ***	24.4%^#^	9.25 ± 1.12 ***	42.2%^###^
FKH 1000	4.93 ± 0.44	6.22 ± 0.44 ***	36.8%^##^	7.32 ± 0.50 ***	61.1%^###ʃ^
Nail gloss (a.u.)					
Placebo	6.48 ± 0.83	6.32 ± 0.67	10.6%	6.77 ± 0.83	11.2%
FKH 500	5.49 ± 0.84	6.71 ± 0.87 ***	37.3%^##^	7.33 ± 0.85 ***	62.2%^###^
FKH 1000	5.80 ± 0.65	7.30 ± 0.79 ***	33.7%^##^	8.38 ± 0.76 ***	67.5%^###^

*Note:* Intragroup* statistical analyses are reported next to raw data (mean ± SEM); intergroup^#^ (actives vs. placebo) and intergroup ʃ (between actives) statistical analyses are reported next to the % variation versus D0 (Δ%45 or Δ%90) and are calculated as average of the variation of each subject. Statistical analyses are reported as follows: *, #, ʃ *p* < 0.05, **, ##, ʃ ʃ *p* < 0.01, and ***, ###, ʃ ʃ ʃ *p* < 0.001.

Similar results were observed in terms of mean percentage of variation versus D0. Both active treatments showed a progressive improvement in skin gloss (+26.5% ± 4.6% and + 25.3% ± 4.7% at D45, +35.8% ± 4.7% and + 43.5% ± 5.2% at D90 for FKH 500 mg and FKH 1000 mg, respectively), hair gloss (+24.4% ± 7.3% and + 36.8% ± 7.5% at D45, +42.2% ± 8.5% and + 61.1% ± 8.1% at D90 for FKH 500 mg and FKH 1000 mg, respectively), nail gloss (+37.3% ± 8.3% and + 33.7% ± 6.4% at D45, +62.2% ± 11.6% and + 67.5% ± 10.4% at D90 for FKH 500 mg and FKH 1000 mg, respectively) that resulted in a significant intergroup improvement achieved by all treatments in comparison to placebo. An intergroup significant difference between the active treatments was also observed for hair gloss at D90.

### 3.5 Epidermis Thickness

**FIGURE 4:**
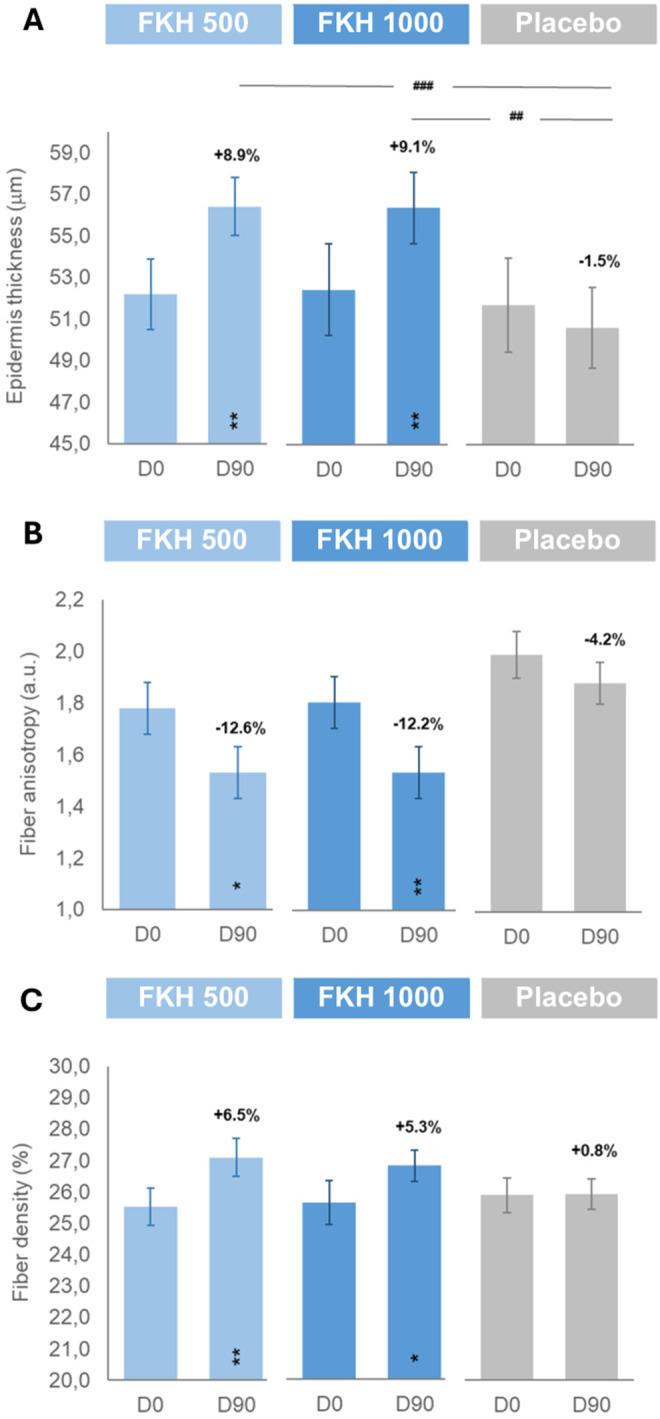
Effect of treatments on epidermis thickness (A), fiber anisotropy (B), and fiber density (C). Results are expressed as mean ± SEM of the respective parameters and as % of variation D90 versus D0 intragroup* and intergroup^#^ (actives vs. placebo as Δ%90); statistical analyses are reported as *^,#^ *p* < 0.05, **^,##^ *p* < 0.01, and ***^,###^ *p* < 0.001.

Whereas placebo treatment did not affect the instrumental values related to facial epidermis thickness (Figure 4A), FKH treatments showed a significant intragroup improvement in this parameter at D90. Mean percentage variations of the active treatments versus D0 (+8.9% ± 2.1% and + 9.1% ± 2.9% for FKH 500 mg and FKH 1000 mg, respectively) also resulted in a significant intergroup difference in epidermis thickness versus the placebo.

### 3.6 Fiber Anisotropy and Fiber Density

Placebo treatment did not affect neither the fiber anisotropy nor the fiber density (Figure 4B,C), whereas FKH treatments showed a significant decrease in fiber anisotropy as well as a significant increase in fiber density at D90 versus D0, indicating a better organization of the fibers in the dermis. Mean percentage of variation versus D0 was similar for both FKH treatments: −12.6% ± 4.0% and − 12.2% ± 4.0% for fiber anisotropy and + 6.5% ± 1.6% and + 5.3% ± 2.2% for fiber density for FKH 500 mg and FKH 1000 mg, respectively, but no significant intergroup differences with respect to the placebo were recorded.

### 3.7 Nail Hardness

All treatments resulted in a progressive increase in the proportion of subjects who reported an improvement of hardness/aspect of their nails (Table 4): 13.8% at D45 and 27.6% at D90 for placebo; 73.3% at D45 and 90.0% at D90 for 500 mg FKH; and 83.9% at D45 and 96.8% at D90 for 1000 mg FKH. Accordingly, VAS score showed a similar increase as follows: a progressive and significant (*p* < 0.001) intragroup increase in VAS score by both active treatments at D45 and D90. Moreover, a significant (*p* < 0.001) intergroup difference was recorded at D45 and D90 between both FKH treatments and with respect to the placebo group. Also, a significant (*p* < 0.05) intragroup increase of nail hardness was noted in the placebo at D90.

**TABLE 4:** Nail hardness.

	D0	D45	D45‐D0	D90	D90‐D0
Nail hardness (score)					
Placebo	4.2 ± 0.2	4.3 ± 0.2	0.1 ± 0.1	4.4 ± 0.3*	0.2 ± 0.1
FKH 500	4.4 ± 0.2	5.1 ± 0.2***^###^	0.7 ± 0.1	5.9 ± 0.2***^###^	1.5 ± 0.1
FKH 1000	4.3 ± 0.2	5.4 ± 0.2***^### ʃ^	1.1 ± 0.1	6.4 ± 0.2***^### ʃ^	2.1 ± 0.2

*Note:* Intragroup* statistical analyses are reported next to raw data (mean ± SEM), and intergroup^#^ statistical analyses (actives vs. placebo) and intergroup ʃ statistical analyses (between FKH treatments) are reported next to raw data (mean ± SEM). Difference versus D0 are calculated as average of the variation of each subject. Statistical analyses are reported as follows: *# ʃ *p* < 0.05, **## ʃ ʃ *p* < 0.01, and ***### ʃ ʃ ʃ *p* < 0.001.

### 3.8 Self‐Assessment Questionnaire

**FIGURE 5:**
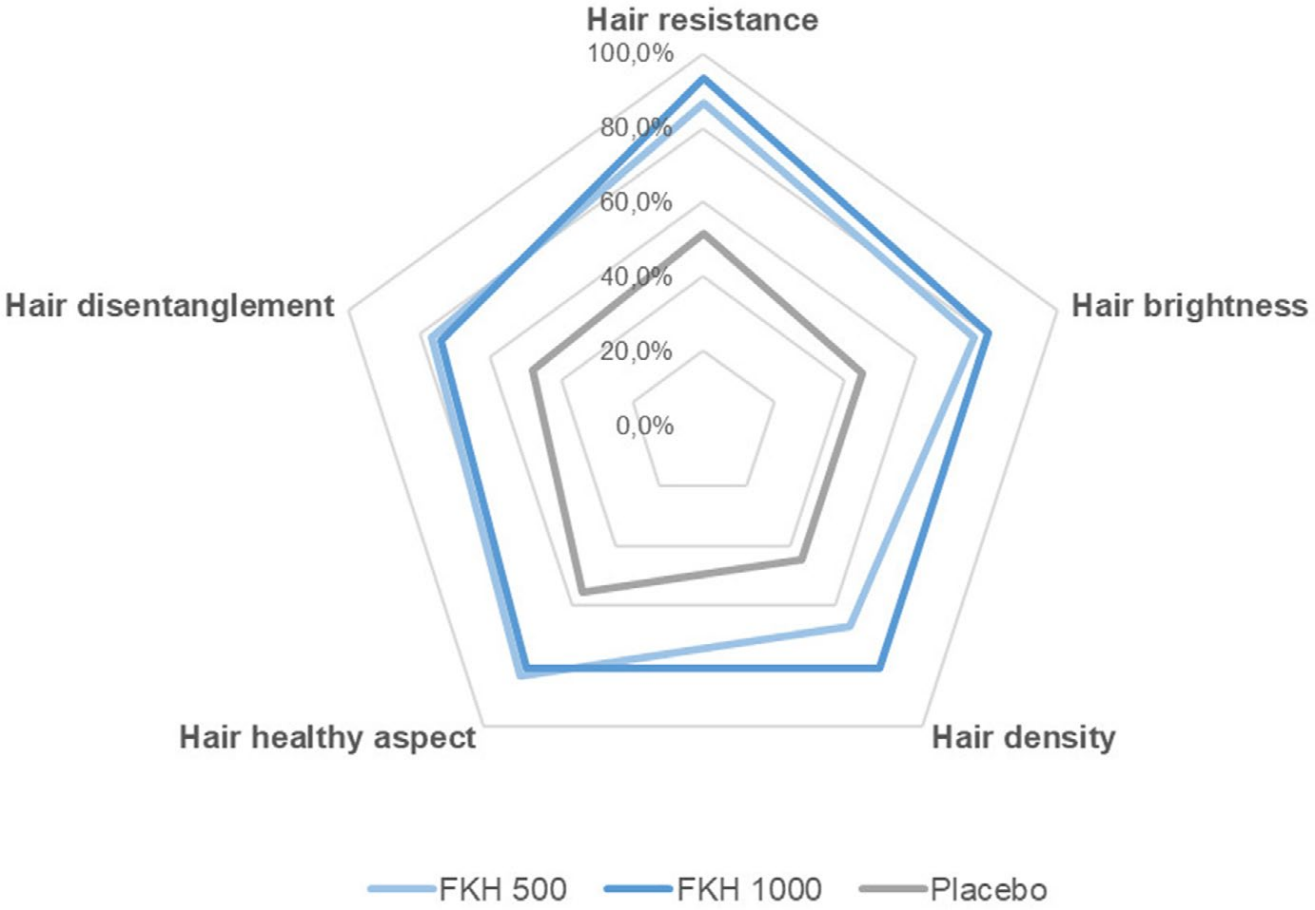
Self‐assessment questionnaire to questions referring to the hair: Mean percentage of positive answers (completely agree + agree).

**FIGURE 6:**
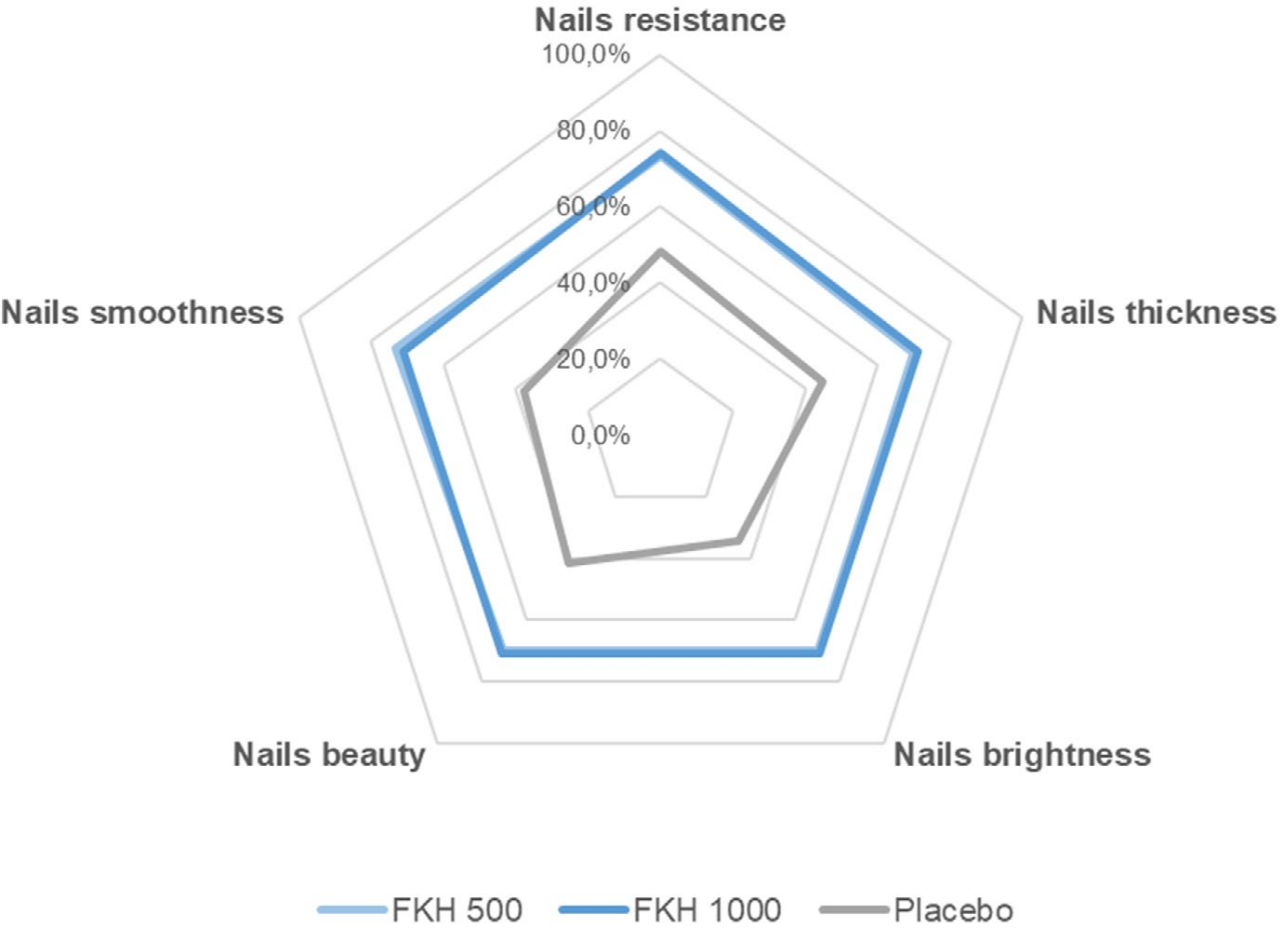
Self‐assessment questionnaire to questions referring to the nails: Mean percentage of positive answers (completely agree + agree).

Results of the self‐assessment questionnaire, filled by all the participants at the end of the study and expressed as positive response (completely agree + agree), indicate very positive judgments for both FKH treatments. Table 5 reports the percentage of positive answers to the 16 questions referring exclusively to skin and indicates that 500 and 1000 mg FKH treatments resulted in a mean of 66.9% and 76.5% of positive answers, respectively, compared to the 46.8% from the subjects receiving the placebo. All results are significantly higher for FKH treatments compared to placebo. The highest percentages of positive answers were expressed for questions related to smoothness, hydration, and nourishment, as well as to the overall appearance of the facial skin. Spider charts showing the distribution of answers to the 5 questions related to hair (Figure 5) and nails (Figure 6) demonstrate the better acceptance of FKH treatments with respect to the placebo. Both FKH treatments were similarly acceptable for nails and with a slightly higher acceptability of FKH 1000 mg treatment for hair.

**TABLE 5:** Self‐assessment questionnaire to questions referring to the skin: Mean percentage of positive answers (completely agree + agree).

No.	Items	FKH 500	FKH 1000	Placebo
01	My facial skin seems smoother?	80.0%	90.3%	55.2%
02	My facial skin seems less wrinkled (eyes, cheeks, mouth)?	76.7%	74.2%	48.3%
03	My facial skin seems more hydrated?	80.0%	90.3%	58.6%
04	My facial skin seems nourished?	80.0%	87.1%	55.2%
05	My facial skin seems firmer?	80.0%	83.9%	51.7%
06	My facial skin tone looks more homogenous?	73.3%	80.6%	48.3%
07	The product reduces facial skin tone irregularities?	63.3%	71.0%	55.2%
08	My facial skin tone looks brighter?	66.7%	83.9%	51.7%
09	The product provides a healthy glow to my face?	66.7%	83.9%	48.3%
10	The product decreases signs of fatigue of my skin?	56.7%	67.7%	41.4%
11	My facial skin is more beautiful?	73.3%	87.1%	55.2%
12	The product helps reducing the appearance of dark spots?	40.0%	54.8%	34.5%
13	The product helps reducing facial skin irritation?	53.3%	64.5%	37.9%
14	Overall appearance of my facial skin is improved?	76.7%	90.3%	51.7%
15	Under eye bags are reduced?^a^	37.5%	42.9%	14.3%
16	My facial skin imperfections are reduced?	66.7%	71.0%	41.4%
	Average	66.9%	76.5%	46.8%

^a^ Only subjects showing eye bags answered to this question (7 subjects belonging to FKH 1000 group, 8 subjects belonging to FKH 500 group, and 7 subjects belonging to placebo group).

**FIGURE 7:**
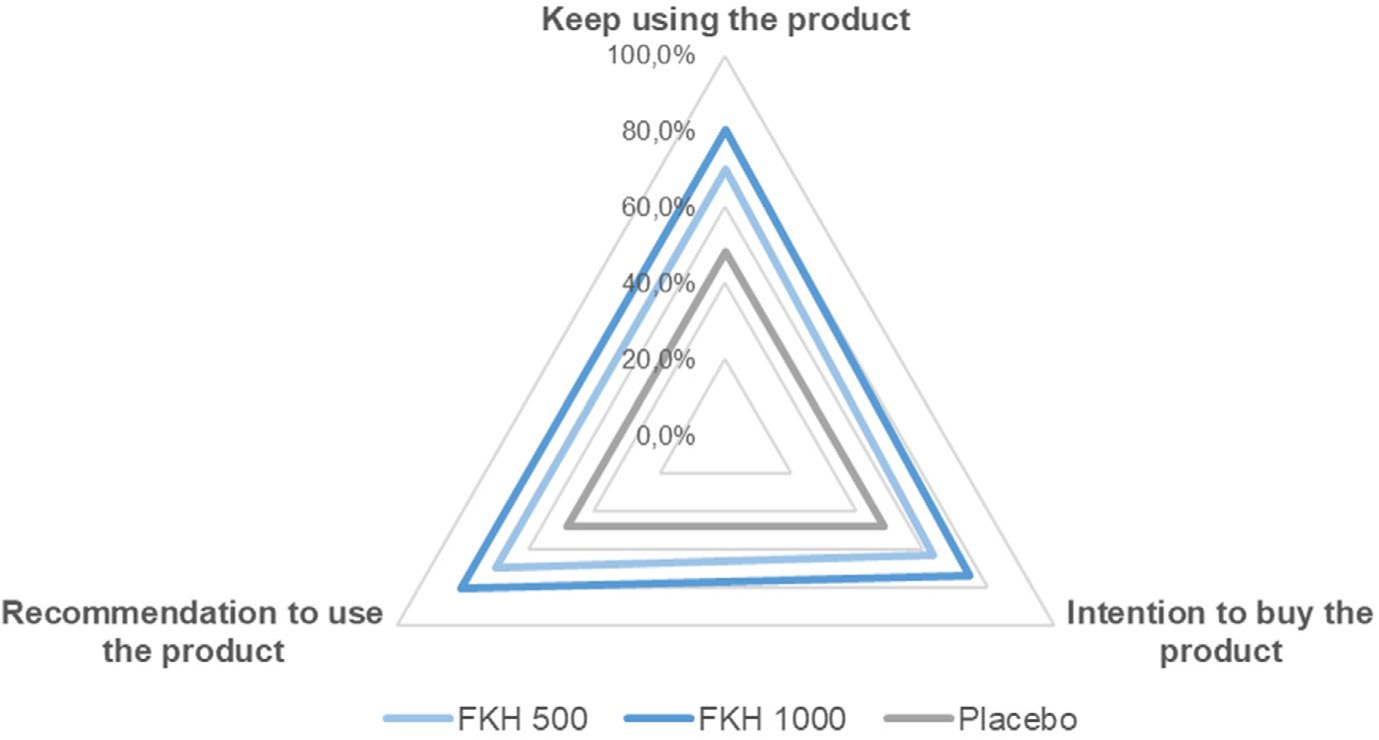
Self‐assessment questionnaire to questions referring to the product satisfaction: Mean percentage of positive answers (completely agree + agree).

The positive results achieved in the previous set of questions referring to the FKH treatments were supported by the satisfaction expressed by the panel in keeping using the product and recommending it (Figure 7).

## 4 Discussion

Hydrolyzed keratin extracted from different biological sources is used as component of food supplements for improving skin, hair, and nail aging. The novelty of the present clinical study is the evaluation of a hydrolysate obtained by a patented extensive hydrolysis process applied on keratin from poultry feathers, which presents a human‐like keratin profile [20]. The resulting hydrolysate is characterized by a specific mix of free L‐amino acids with a very high content of L‐free amino acids (≥ 83.5%), providing a unique ingredient for food supplements, which differs from other commercial keratin hydrolysates consisting of mixtures of unidentified macro peptides, generally combined with other components such as vitamins and minerals. The results achieved in this clinical study suggest an interesting hypothesis about the mode of action of FKH that could be related to the qualitative and quantitative profile of FKH resulting from a specific extensive hydrolysis process. These results, related to skin anti‐aging properties and to hair and nail strengthening effects, were also directly perceived by the subjects participating in the study (self‐assessment questionnaire, Table 5, Figures 5 and 6).

Free amino acids provided by FKH are easy to digest and are rapidly absorbed. Consequently, they can participate in different biological pathways, which may explain the skin benefits observed in this study. In the healthy gut, dietary amino acids are efficiently taken up by different amino‐acid transporters in the enterocytes of the proximal jejunum with a lesser contribution from the ileum [21]. The large intestine also expresses amino acid transporters and mediates the uptake of amino acids derived from bacterial metabolism and endogenous sources. It has been estimated that 2%–7% of the daily protein uptake enters the large intestine suggesting a potential prebiotic effect [22]. At the same time, as the prebiotic properties of other types of bioactive components such as polyphenols are being discovered, it can be hypothesized that amino acids may behave similarly. A recent publication shows that aging can influence gut microbiome and accelerate age‐related skin changes through an increase in “inflammaging,” but also by promoting senescent cell accumulation and compromising the host's immune response [23]. In this manner, amino acids may modulate the gut microbiota composition and promote the growth of beneficial bacteria while counteracting the development of pathogens. Amino acids may also be used by selective intestinal bacteria to be transformed into metabolites with a positive impact on host health including skin [23, 24]. In addition, chemical diversity of amino acids within FKH may lead to the production of multiple bacterial metabolites with broad biological activity. We can summarize all these biological activities under the term of “aminobiotics,” which refers to the functional role of some amino acids as prebiotics [24]. Such promising mechanism of action needs to be carefully investigated, for example, through pharmacokinetic studies with an evaluation of stool composition that could help identify some potential modifications of gut microbiota following FKH absorption by humans.

Besides this innovative mechanism, the role of amino acids as building blocks of different molecules in the body remains of interest to explain the observed clinical benefits of FKH. The proper balance of amino acids level should be maintained to preserve good skin conditions. Age‐related alterations in amino acids metabolism may impair the body's capacity to regenerate skin cells efficiently, which can contribute to the appearance of aging signs [25].

Therefore, supplementation with free amino acids could represent a novel anti‐aging strategy to maintain skin homeostasis through effective cell renewal and protein synthesis including the most abundant fibrous proteins of the skin like keratins, collagen, and elastin which play a pivotal role in the biomechanical properties of the skin [26].

In the skin, amino acids play different roles. For example, Natural Moisturizing Factor (NMF), essential to desquamation, plasticity, and strength of skin barrier, is roughly composed of 40% of free amino acids [27]. Therefore, it can be hypothesized that amino acids brought by blood circulation may enter the NMF composition and hence help to maintain skin homeostasis and to improve skin hydration. In the same way, aquaporins, which are membrane proteins that serve as channels and regulate water flow in and out of the cells, may partner with amino acids to facilitate smooth water conduction among skin cells [28]. Consequently, oral free amino acids can help the cells in the stratum corneum to retain moisture through NMF production while supporting aquaporins functionalities, which, in turn, promotes the overall moisture content of the skin.

Sulfur‐containing amino acids, like cystine, which are found in FKH, are the most bioavailable sulfur sources for the human body. Sulfur is the third most abundant mineral in the body and is mainly present in connective tissues such as skin, tendons, and ligaments where it is essential for the cross‐linking of the extracellular matrix proteins. Indeed, disulfide bonds are key to the strong, yet flexible properties of the connective tissues [29, 30]. Sulfur amino acids contribute also substantially to the maintenance and integrity of the cellular systems by influencing cellular redox status and with the capacity to detoxify toxic compounds, free radicals, and reactive oxygen species [30, 31].

In the body, amino acids can also enhance antioxidant status. Glutathione (GSH) is synthesized from the amino acids glutamate, cysteine, and glycine so that increasing the supply of L‐Cysteine or its precursors enhances L‐GSH synthesis [30]. A previous clinical trial showed that a food supplement containing L‐Cystine associated with L‐Glutathione could mediate a skin whitening/lightening effect through an increase of GSH synthesis [32]. The contribution of FKH to cystine, glutamate, and glycine supply may enhance glutathione synthesis in the body and improve the management of redox reactions. Supplementation with amino acids may support GSH status hence protecting the body, including the skin, from oxidative stress generated by UV exposure, for example. Recently, it has been reported that amino acids not only are the building blocks of proteins but also work as signaling molecules regulating multiple biological processes [33]. This signal effect has broad implications that may partly explain the above‐described clinical effects.

Measures on skin cells (fibroblasts or keratinocytes) might give additional information on the underlying mechanisms such as cellular viability, possible ROS reduction or decrease of endoplasmic reticulum stress (via the UPR pathway). Hypotheses including effects on senescence and apoptosis mechanisms should also be investigated with additional in vitro trials and clinical studies. A recent study reported the effects and mechanisms of amino acids on skin aging signs and highlighted the role of arginine and glycine on skin barrier, wound healing, or as antioxidant preventing skin degradation [34]. Formerly, a supplementation combining glycine and n‐acetylcysteine showed positive effects on aging in older adults with improvements on GSH deficiency, oxidative stress, mitochondrial function, inflammation, body function, and markers of aging [35].

The number of participants and the focus on women in this study appear to be a limitation. Thus, the results of the present study investigating the role of FKH as a dietary supplement for skin health and beauty should be confirmed by a larger clinical study including both men and women with a wider age group. Different hypotheses regarding the mechanism of action of FKH have been proposed, but it is still unclear how FKH, as free amino acids supplement, helps to improve skin quality. The design of a new study should investigate the biological roles of free amino acids for the skin, by including, for example, stripping or skin biopsies to identify biological variations and new metabolic pathways to explain the clinical results achieved. Metabolic mapping could also be performed to identify the biological pathways involved in skin hydration, anti‐aging effects, or skin homeostasis as a whole, as observed in this study. This will also help to identify the metabolites involved in these clinical benefits and inform further product development and new applications.

## 5 Conclusion

A 3‐month oral administration of both 500 and 1000 mg daily doses of FKH significantly mitigated aging signs in a panel of adult women. A progressive and significant improvement was achieved on instrumental parameters related to skin profilometry, including roughness, wrinkles features (length, depth, and area), as well as on epidermis water content and thickness. All these results suggest a positive remodeling of the skin epidermis. A similar significant effect was recorded on skin elasticity and maximum elongation which are representative of a structural modification of the extracellular matrix resulting in a more functional skin dermis. FKH treatments significantly improved skin anisotropy and skin density compared to their basal values, although such increments were not significantly different from placebo, presumably due to the smaller number of subjects (*n* = 20) involved in the measurement of these parameters. Skin is supposed to become more anisotropic with age [18, 36] and the evaluation of such a parameter is considered as a novel approach to better understand skin biomechanical properties and their modifications in time.

FKH treatments, at 500 or 1000 mg/day, also achieved significant improvement in various hair and nail parameters, such as gloss, nail hardness, and general aspect, confirming the preliminary results of a previous clinical trial [9, 10] in which a 1000 mg/day dosage of FKH including vitamins and minerals was shown to improve hair density, percentage of hair in anagen phase, hair and nail gloss, and nail growth [10]. The current study extends these results and demonstrates a significant effect of FKH, commercialized under the brand name Kera‐Diet (KeraGLO in the United States) as a sole ingredient, on skin, hair, and nails with a dose of 500 mg/day. The instrumental measurements were also supported by the positive judgments expressed by the subjects who received the active treatments.

## Author Contributions

**Vincenzo Nobile**, **Renaud Sergheraert**, and **Jean‐Philippe Soulard:** conceptualization. **Francesco Tursi**, **Enza Cestone**, and **Vincenzo Nobile:** methodology. **Vincenzo Nobile**, **Francesco Tursi**, **Enza Cestone**, and **Ileana De Ponti:** validation. **Vincenzo Nobile** and **Francesco Tursi:** formal analysis. **Enza Cestone:** investigation. **Vincenzo Nobile** and **Ileana De Ponti:** resources. **Vincenzo Nobile** and **Francesco Tursi:** data curation. **Francesco Tursi** and **Jean‐Philippe Soulard:** writing – original draft preparation. **Vincenzo Nobile,** **Francesco Tursi** and **Jean‐Philippe Soulard:** writing – review and editing. **Vincenzo Nobile:** visualization. **Vincenzo Nobile** and **Ileana De Ponti:** supervision. **Ileana De Ponti:** project administration. **Ileana De Ponti:** funding acquisition. All authors have read and agreed to the published version of the manuscript.

## Conflicts of Interest

The authors declare no conflicts of interest.

## Data Availability

The data that support the findings of this study are available from the corresponding author upon reasonable request.
